# Innovative resource-limited endoscopy simulator for skill development

**DOI:** 10.1055/a-2767-0666

**Published:** 2026-01-15

**Authors:** Khurum Hakeem, Yahya Al Hammada, Naif Al-Hakmani, Khalid AlNaamani, Samer Al-Dury

**Affiliations:** 1 105565 Freeman Hospital, Newcastle upon Tyne, United Kingdom; 2 3570 Sahlgrenska Academy, University of Gothenburg, Gothenburg, Sweden; 3 Muscat Endoscopy Academy, Endoscopy Unit, Division of Gastroenterology and Hepatology, Medical City for Military and Security Services, Muscat, Oman; 4 3570 School of Public Health and Community Medicine, Institute of Medicine, University of Gothenburg, Gothenburg, Sweden; 5 60200 Department of Gastroenterology and Hepatology, Ghent University Hospital, Ghent, Belgium

We present a do-it-yourself endoscopy simulator constructed from low-cost, widely available materials. Mechanical, box-based simulators are recognized in recent ESGE guidance as accessible tools supporting early skill acquisition within structured endoscopy training 1 . This project aligns with these recommendations by offering a simple, affordable model that enables repeated practice in a controlled environment.

**Fig. 1:**
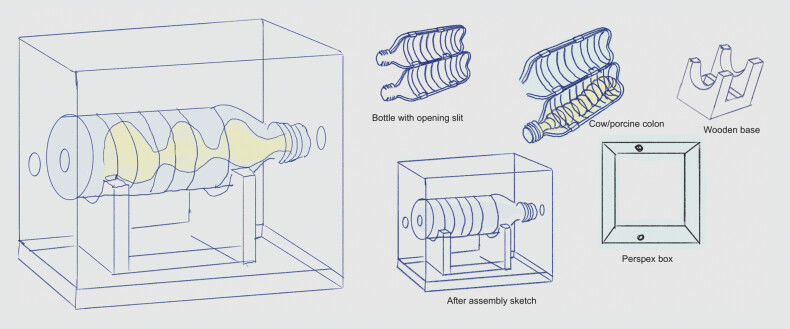
Schematic of endoscopy simulator the drawing shows the training simulator used in this project. A Perspex enclosure houses a clear bottle with an intestine on a wooden mounting block.

The simulator used in this project ( Fig. 1 ) consists of a Perspex plastic enclosure with entry ports and interchangeable internal inserts designed to mimic the behavior of the colon, including key anatomical landmarks. In the accompanying video ( Video 1 ), we demonstrate assembly as well as performance of core exercises such as mucosal inspection, improved reaction time, and a range of therapeutic maneuvers. The model enables repeated practice of scope handling, instrument use, and fine tip control without patient risk.

Low-cost Perspex-box simulator showing navigation, mucosal assessment, injection, hot snaring using endoscopic mucosal resection (EMR) principles, and stepwise endoscopic submucosal dissection (ESD) including incision, trimming, and dissection.Video 1

It supports both foundational skill acquisition for trainees and refinement of advanced techniques for experienced endoscopists. Its educational value lies in allowing deliberate, self-paced practice in a safe, controlled setting with immediate visual feedback and the ability to escalate task complexity.

Published work 2 further supports the utility of box-based simulators for novice endoscopists to acquire basic technical skills and emphasizes the role of simulation within structured training curricula. By sharing practical construction details and a concise technique demonstration, this video aims to broaden access to simulation-based training, particularly in resource-limited environments, and contribute to more equitable endoscopy education.

Endoscopy_UCTN_Code_TTT_1AU_2AB
